# Galectin-3-Binding Protein Inhibits Extracellular Heparan 6-*O*-Endosulfatase Sulf-2

**DOI:** 10.1016/j.mcpro.2024.100793

**Published:** 2024-06-01

**Authors:** Aswini Panigrahi, Julius Benicky, Reem Aljuhani, Pritha Mukherjee, Zora Nováková, Cyril Bařinka, Radoslav Goldman

**Affiliations:** 1 Department of Oncology, Lombardi Comprehensive Cancer Center, Georgetown University, Washington, District of Columbia, USA; 2 Clinical and Translational Glycoscience Research Center, Georgetown University, Washington, District of Columbia, USA; 3 Department of Biochemistry and Molecular & Cellular Biology, Georgetown University, Washington, District of Columbia, USA; 4 Institute of Biotechnology of the Czech Academy of Sciences, BIOCEV, Vestec, Czech Republic

**Keywords:** Sulf-2, affinity purification, mass spectrometry, protein-protein interaction, HNSCC, spheroid, sulfatase, enzyme, LC-MS/MS

## Abstract

Human extracellular 6-*O*-endosulfatases Sulf-1 and Sulf-2 are the only enzymes that post-synthetically alter the 6-*O* sulfation of heparan sulfate proteoglycans (HSPG), which regulates interactions of HSPG with many proteins. Oncogenicity of Sulf-2 in different cancers has been documented, and we have shown that Sulf-2 is associated with poor survival outcomes in head and neck squamous cell carcinoma (HNSCC). Despite its importance, limited information is available on direct protein–protein interactions of the Sulf-2 protein in the tumor microenvironment. In this study, we used monoclonal antibody (mAb) affinity purification and mass spectrometry to identify galectin-3-binding protein (LG3BP) as a highly specific binding partner of Sulf-2 in the conditioned media of HNSCC cell lines. We validated their direct interaction *in vitro* using recombinant proteins and have shown that the chondroitin sulfate (CS) covalently bound to the Sulf-2 influences the binding to LG3BP. We confirmed the importance of the CS chain for the interaction by generating a mutant Sulf-2 protein that lacks the CS. Importantly, we have shown that the LG3BP inhibits Sulf-2 activity *in vitro* in a concentration-dependent manner. As a consequence, the addition of LG3BP to a spheroid cell culture inhibited the invasion of the HNSCC cells into Matrigel. Thus, Sulf-2 interaction with LG3BP may regulate the physiological activity of the Sulf-2 enzyme as well as its activity in the tumor microenvironment.

## Highlights

• Galectin-3-binding protein (LG3BP) is a specific binding partner of Sulf-2.
• LG3BP non-competitively inhibits Sulf-2 enzymatic activity *in vitro*.
• In spheroid model LG3BP inhibits invasion of the HNSCC cells into Matrigel.

## Graphical Abstract

**Figure:**
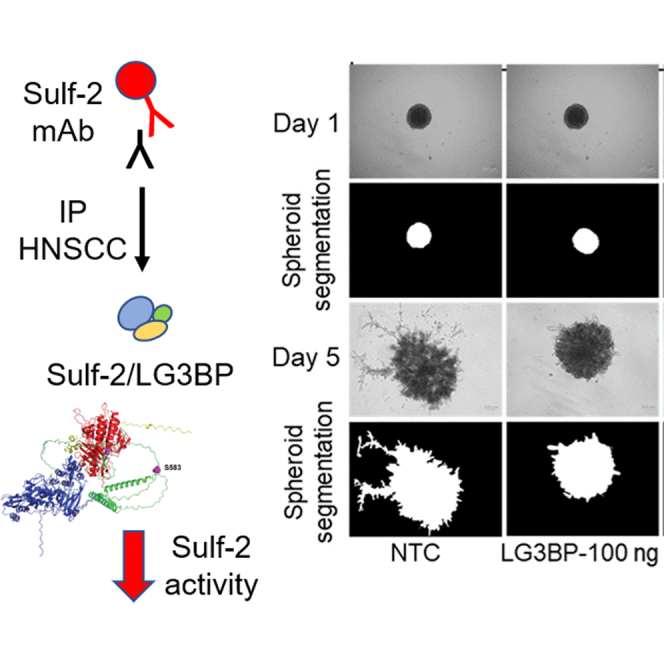

## In Brief

Monoclonal antibody affinity purification and mass spectrometry were used to identify galectin-3-binding protein (LG3BP) as a binding partner of Sulf-2 in HNSCC cell lines conditioned media. Sulf-2 chondroitin sulfate side chain influences the binding to LG3BP, and we confirmed its importance by generating a mutant Sulf-2 protein. We have shown that the LG3BP non-competitively inhibits Sulf-2 enzymatic activity *in vitro*, and consequently addition of LG3BP to a spheroid cell culture inhibited the invasion of the HNSCC cells into Matrigel.

Human extracellular 6-*O*-endosulfatases Sulf-1 and Sulf-2 are homologous enzymes that exhibit arylsulfatase activity and highly specific endoglucosamine-6-sulfatase activity (1, 2, 3). These two are the only enzymes that post-synthetically alter the 6-*O* sulfation of heparan sulfate proteoglycans (HSPG) which affects binding to multiple ligands including VEGF, FGF, HGF, fibronectin, or certain chemokines (4, 5, 6, 7, 8, 9, 10). These interactions regulate many impactful signaling events and fundamentally modulate various developmental processes or pathological activities in the tumor microenvironment. Studies from our and other laboratories have shown the upregulation of Sulf-2 in various cancer types and its negative impact on patient survival (11, 12, 13, 14, 15, 16, 17, 18). Thus, the Sulf-2 enzyme is expected to serve as essential regulatory elements in HS-dependent developmental and pathophysiological processes (19, 20).

Sulf-2 is a secretory enzyme that has been characterized by us (21) and others (9, 20, 22). The enzyme is extensively N-glycosylated (21) and processed by furin-cleavage to a mature form consisting of a disulfide-bound pair of subunits (2). The highly conserved N-terminal subunit contains the catalytic active site carrying a formylglycine residue generated from a cysteine by a dedicated SUMF1 processing enzyme (23, 24, 25). Recent studies show that the C-terminal hydrophilic domain of Sulf-2 carries a chondroitin sulfate chain at position S583, which distinguishes it from the Sulf-1 enzyme (26). A schematic of Sulf-2 protein showing domains and post-translational modifications (PTMs) is shown in Supplemental Fig. S1. The activity of Sulf-2 is regulated by the chondroitin sulfate (20, 26) and by its N-glycosylation (21). It is also known that the catalytic activity is sensitive to ionic influences and buffer composition (2, 21). However, virtually nothing is known about the regulation of the enzymatic activity by interaction with other proteins.

We anticipated that the interactions of the Sulf-2 enzyme with other proteins in the extracellular space regulate its function. To test our hypothesis, we performed monoclonal antibody (mAb) affinity purification of the native Sulf-2 and its interacting partners from the conditioned media of head and neck squamous cell carcinoma (HNSCC) cell lines. We identified and validated the highly specific binding of Sulf-2 to galectin-3-binding protein (Gene: LGALS3BP, UniProtKB Q08380). Furthermore, using *in vitro* enzymatic and cell culture assays, we showed that the interaction inhibits Sulf-2 enzymatic activity and modifies invasion of cancer cells into matrigel.

## Experimental Procedures

### Materials

Amicon Ultra-15 (Millipore), Dynabeads M-280 sheep anti-Mouse IgG (Invitrogen), Anti-Sulf-2 monoclonal antibodies (QED Bioscience), Anti-LG3BP monoclonal antibody (Proteintech), Mouse IgG1 k Isotype control (eBioscience), BSA (NEB), Recombinant Sulf-2 protein (produced in house), Recombinant human galectin-3-binding protein (R&D Systems, and produced in-house), QuikChange Lightning Site-Directed Mutagenesis Kit (Agilent Technologies), 25 kDa polyethyleneimine (Polysciences), Custom heavy isotope-labeled Sulf-2 peptide (Biosynth), Trypsin/Lys-C protease (Thermo Scientific), Empore C18 cartridge (CDS Analytical), Nano-flow C18 columns, and other reagents and chemicals were obtained from Thermo Scientific.

### Cell Culture and Conditioned Media Collection

Two HNSCC cell lines, SCC35 and CAL33, were cultured as described previously (27). Briefly, the cells were grown to 70% confluency in DMEM containing 10% FBS, following which they were washed with and incubated in pre-warmed (37^o^C) serum-free conditioned media for 24h at 37^o^C. The conditioned media were collected and non-ionic detergent Tween-20 was added to a final concentration of 0.01%. Cells and any cell debris were removed by two-step centrifugations, 800*g* for 10 min at 4^o^C, followed by 4000*g* for 10 min at 4^o^C. The supernatant was concentrated to 50x using Amicon Ultra-15 Centrifugal Filters (30 kDa cutoff), and further centrifuged at 16,200*g* for 15 min to remove any aggregates. The cleared concentrated sample was used in our immunoprecipitation assays.

### Production of Recombinant Sulf-2 and LG3BP Using a Lentiviral Expression System

SULF2 ORF including C-terminal Myc-His tag was subcloned from its source vector (Addgene # 13003) to lentiviral transfer vector pHR-CMV-TetO2_3C-Twin-Strep (Addgene # 113883) (28). Galectin-3-binding protein ORF was subcloned from its source vector (Origene # RC204918) into bicistronic lentiviral transfer vector pHR-CMV-TetO2 3C-TwinStrep-IRES-EmGFP vector (Addgene, # 113884) modified to introduce 6xHis tag downstream of a cleavable C-terminal TwinStrep tag. The above transfer vectors were co-transfected with envelope (pMD2.G, Addgene) and packaging (psPAX2, Addgene) plasmids to Lenti-X HEK293T cells (Takara) to generate lentiviral particles for transduction of the target cells as described in detail previously (28). The His-tagged Sulf-2 (Q8IWU5) and LG3BP (Q08380) proteins were purified using a nickel affinity column as described earlier (21). The SDS-PAGE protein profile of the purified LG3BP sample is shown in Supplemental Fig. S2.

### Generation of Mutant Sulf-2 Protein without Chondroitin Sulfate

We described previously (21) the production of a recombinant Sulf-2 protein using a stably transfected HEK293F cell line. We used the same expression system to generate a cell line expressing a Sulf-2 protein with a Ser583Ala (S583A) point mutation, which eliminates the chondroitin sulfate (CS) attachment site (position) from the expression vector pcDNA3.1/Myc-His(−)-HSulf-2 bearing human Sulf-2 cDNA (a gift from Steven Rosen, Addgene plasmid # 13004) (2). The Sulf-2 S583A mutation was generated using mutagenic primers 5′- GGCCTCCAGTGCCAGCGAAGTCCCCACCAT-3’ (sense) and 5′-ATGGTGGGGACTTCGCTGGCACTGGAGGCC-3′, and site-directed mutagenesis kit, according to the manufacturer’s instructions. The construct was transfected into HEK293F cells using linear 25 kDa polyethylenimine at nitrogen-to-phosphate (N/P) ratio 20. Stable transfectants were generated by antibiotic resistance selection using 500 μg/ml G418.

### Experimental Design and Statistical Rationale

Proteomics experiments of Sulf-2 mAb-affinity purified samples were conducted using conditioned media of two different HNSCC cell lines, SCC35 and CAL33. Three biological replicates of each cell line were performed to capture the reproducibility of interacting protein target identification. Validation of protein-protein interaction was performed using reciprocal mAb-affinity purification of Sulf-2, and in parallel its binding partner LG3BP from two recombinant Sulf-2 expressing cell lines Sulf-2 HEK293F, and Lenti-Sulf-2 HEK293T. The proteins in the affinity-purified samples were identified by LC-MS/MS analysis as described below. Candidate interacting proteins were deduced based on gene ontology functional enrichment analysis compared to negative controls.

The effect of a Sulf-2 PTM, chondroitin sulfate side-chain at position 583 on protein–protein interaction was studied by chondroitinase treatment of recombinant Sulf-2 HEK293F conditioned media followed by determination of the relative amount of Sulf-2 and LG3BP in anti-LG3BP mAb-affinity purified sample by targeted LC-MS/MS-PRM analysis. We also determined the relative ratio of Sulf-2 and LG3BP in anti-Sulf-2 mAb-affinity purified sample from the conditioned media of three different recombinant cell lines, Sulf-2 HEK293F, Lenti-Sulf-2 HEK293T, and Sulf-2-S583A mutant HEK293F cell lines, where the level of Sulf-2 chondroitination varies. Three technical replicates were performed for each experiment, and unpaired *t* test or one-way ANOVA analysis was performed using GraphPad Prism software (v 10.1.1) to determine the statistical significance between the datasets (*p* < 0.05 considered significant), and the observed results are indicated in the figure by an asterisk (∗∗∗∗ denotes *p* < 0.0001).

The effect of LG3BP on Sulf-2 enzymatic activity was measured using an HPLC-UV assay. Half-maximal inhibitory concentration (IC50) of LG3BP on Sulf-2 activity, and Sulf-2 enzymatic kinetics with varying concentrations of LG3BP, and substrate were determined. All assays were performed in triplicate. The effect of added LG3BP on Sulf-2 activity was also measured in the spheroid cell culture model using SCC35 and HNCAF61137 cells. Six replicates per condition were carried out and GraphPad Prism Software (v 10.1.1) was used to calculate Mean and SD. Two-way ANOVA with Tukey was used to determine statistical significance.

### Immunoprecipitation of Native Sulf-2 and Interacting Proteins

The native Sulf-2 protein was immunoprecipitated from the concentrated conditioned media of the Cal33 and SCC35 HNSCC cell lines using anti-Sulf-2 monoclonal antibodies (mAbs 5D5 and 5C12). The following steps were carried out at 4 °C (a schematic of the IP method is shown in Supplemental Fig. S3). The mAbs (5 μg each diluted in 500 μl of IP buffer: 1x PBS, 0.1% Tween 20) were incubated with 100 μl of anti-mouse IgG coated magnetic beads (Dynabeads M-280) and 10 μg BSA for 1 h with rotation. Beads incubated with mouse IgG isotype served as negative control. The beads were washed thrice with IP buffer (1 ml each step) and the antibody-bound beads were incubated with 500 μl of the concentrated SCC35 or CAL33 conditioned media sample in parallel plus 500 μl IP buffer for 1h with rotation. Unbound materials were removed by washing the beads three times with the IP buffer (1 ml each step, 5 min each incubation, with rotation). The beads were further washed thrice with 1x PBS to remove residual detergent from the sample (for ease of downstream mass spectrometry analysis). The bound proteins were eluted by incubating the beads in 10 mM Glycine pH 2.6 and neutralized with Tris buffer. The eluates were processed for mass spectrometry analysis as described below.

### Immunoprecipitation of Recombinant Sulf-2 and Interacting Protein From Conditioned Media

We collected cleared conditioned media from HEK293F or HEK293T cells expressing recombinant Sulf-2 protein, and HEK293F cells expressing S583A-Sulf-2 mutant protein. The media was concentrated 5x using a 30 kDa cut-off membrane, and cleared by centrifugation as described above. The Sulf-2 and its interacting protein(s) were pulled down from these samples using anti-Sulf-2 monoclonal antibodies as above. In parallel, LG3BP and associated proteins were pulled down using anti-LG3BP mAb, and the IP steps were carried out as above using 5 μg mAb. We also treated Sulf-2 HEK293F concentrated media with 0.2 U of chondroitinase ABC (Sigma) for 1 h at 37 °C, and performed the affinity purification using anti-LG3BP mAb. The bound proteins were eluted with 10 mM glycine as above and used for LC-MS/MS or Western blot analysis.

### Reciprocal Co-IP Analysis

Co-immunoprecipitation experiments were performed using purified recombinant proteins to validate their interaction. Briefly, anti-mouse magnetic beads (50 μl suspension) were incubated with anti-LG3BP monoclonal antibody in IP buffer and washed as above. Then the beads were incubated with purified recombinant LG3BP (R&D Systems) and washed thrice with IP buffer. The LG3BP bound beads were incubated with purified recombinant Sulf-2 proteins, and washed with IP buffer to remove unbound proteins. Simultaneously, a reciprocal pulldown experiment was performed where the beads were first incubated with Sulf-2 mAb, then with purified Sulf-2 protein, followed by LG3BP protein. The bound proteins were eluted as above, and analyzed by mass spectrometry as described below.

### SDS-PAGE and Western Blot Analysis

The proteins in the immunoprecipitated sample were denatured in SDS-PAGE loading buffer with or without DTT and were separated by SDS-PAGE using NuPAGE 4 to 12% polyacrylamide gels. Western blot was performed with Sulf-2 (1:2500 dilution) and LG3BP mAbs (1:5000 dilution).

### LC-MS/MS Analysis

The proteins in immunoprecipitated samples were reduced/alkylated by treatment with TCEP (10 mM) and iodoacetamide (6 mM), followed by in-solution proteolytic digestion in Barocycler with Lys-C/Trypsin (200 ng in 100 μl volume). The resulting peptides were desalted using a C18 cartridge and analyzed by mass spectrometry using a Dionex RSLCnano–Orbitrap fusion lumos LC-MS platform configured with Acclaim PepMap 100 trap column (75 μm x 2 cm, C18, 3 μm, 100A), and PepMap RSLC C18 EASY-spray analytical column (75 μm x 15 cm, 3 μm, 100A). The peptides were separated using a 60-min acetonitrile gradient at a flow rate of 0.3 μl/min; solvent A (0.1% formic acid in water), solvent B (0.1% formic acid in acetonitrile), 0 to 5 min peptide load to trap column at 2% B (valve switched at 5 min to direct the flow to analytical column), 5 to 35 min 5 to 25% B, and 35 to 45 min 25 to 90% B, 45 to 53 min 90%B, 53 to 60 min 2% B. The Orbitrap MS parameters were set at nanospray voltage of 2.0 kV, capillary temperature 275 °C, MS1 scan range m/z 400 to 1800, resolution 120,000, RF lens 60%, and MS2 intensity threshold 2.0 × 10^4^, included charge states 2 to 7, and dynamic exclusion for 15s. Data-dependent HCD MS/MS spectra were collected at 7500 resolution with 30% collision energy.

### LC-MS/MS Data Analysis

The acquired mass spectrometry data were searched against combined UniProtKB human (reviewed 20,360 entries) and bovine (reviewed 6044 entries) databases (downloaded April 2024), and common contaminants database (244 entries) using Proteome Discoverer software (v 2.3). Search criteria included full digestion with trypsin, a maximum of two internal missed cleavage sites, peptide length 6 to 144, precursor mass tolerance of 20 ppm, and fragment mass tolerance of 0.5 Da. Static modification for C (carbamidomethyl +57.021), and dynamic modification for M (oxidation +15.995), and dynamic protein N-terminal acetyl modification (+42.011) were specified. The search was performed using the Sequest HT search engine. The search output was filtered with the Percolator algorithm at an FDR of 0.01. Furthermore, we only considered proteins identified with two or more peptide matches and an expected q-value of zero for downstream analysis.

### Targeted LC-MS/MS-PRM Analysis

The relative abundance of Sulf-2 in chondroitinase-treated anti-LG3BP mAb IP samples was determined by targeted mass spectrometry using the Tier three method. Briefly, the samples were digested with trypsin and targeted MS/MS was performed in triplicate to collect product ions of Sulf-2 peptides AEYQTACEQLGQK (*m/z* 763.3512, *z* 2), WQCVEDATGK (*m/z* 597.2664), and LG3BP peptides SQLVYQSR (490.7616) and YSSDYFQAPSDYR (*m/z* 799.8415, *z* 2). The peptides were selected based on the results from our discovery studies. The LC gradient was 1h and the parameters were as above, the HCD product ion spectra of the selected precursors were collected at 1.6 *m/z* isolation window, 30% collision energy, and 15k orbitrap resolution. Xcalibur Quan Browser software was used to extract the peak area of the peptides by sum of three product ions; AEYQTACEQLGQK (y7 862.4087, y8 933.4458, y9 1034.4935), WQCVEDATGK (y6 620.2886, y7 719.3570, y8 879.3877), SQLVYQSR (y4 553.2729, y5 652.3413, y6 765.4254), and YSSDYFQAPSDYR (y5 637.2940, y7 836.3897, y8 983.4581). The peak area of both peptides from the same specific protein was averaged, and the Sulf-2 area was divided by the LG3BP area, to assess the relative change in bound Sulf-2 protein in chondroitinase-treated *versus* untreated pulldown samples. An unpaired *t* test was performed to determine the statistical significance between the datasets.

The anti-Sulf-2 mAb IP samples prepared in parallel from HEK cell conditioned media (recombinant Sulf-2 HEK293F, Lenti-Sulf-2 HEK293T, and Sulf-2-S583A mutant HEK293F) were digested and spiked with five fmol/μl of a heavy (C^13^, N^15^) labeled Sulf-2 internal peptide AEYQTACEQLGQK∗. Targeted Tier 3 LC-MS/MS-PRM assay was performed as above to collect the product ions of native Sulf-2 peptide AEYQTACEQLGQK (*m/z* 763.3512, *z* 2), and heavy Sulf-2 peptide AEYQTACEQLGQK∗ (*m/z* 767.3583, *z* 2), and LG3BP peptides SQLVYQSR (490.7616), and YSSDYFQAPSDYR (*m/z* 799.8415, *z* 2). Xcalibur Quan Browser software was used to extract the peak area of the peptides by the sum of three product ions as above, and AEYQTACEQLGQK∗ (y7 870.4229, y8 941.4600, y9 1042.5077). The peak areas of both LG3BP peptides were averaged, divided by the peak area of spiked Sulf-2 peptide, and converted to percent to reflect normalized data across the runs. To assess the relative LG3BP protein amount compared to Sulf-2 between sample sets, LG3BP/Sulf-2 ratio was calculated, and one-way ANOVA analysis was performed to determine the statistical significance.

### *In silico* Modeling of Sulf-2 Structure and Sulf2/LG3BP Interaction

A structural model of human Sulf-2 protein was predicted using AlphaFold2 server (29) and visualized using PyMOL (v.3.0.2) with the APBS electrostatics plugin. We generated 3D models of free Sulf-2, as well as the Sulf-2/LG3BP complex.

### Sulf-2 enzymatic Activity Assay

The effect of LG3BP on Sulf-2 enzymatic activity was measured using 2S2-6S4 substrate, a recently described HPLC-UV assay (21). Briefly, LG3BP (R&D Systems) at a 2-fold dilution series (concentration range of 2–16 μg/ml) was preincubated with a fixed amount of Sulf-2 (0.4 μg/ml) for 30 min before addition of 100 μM 2S2-6S4 substrate. BSA was added in place of LG3BP (at the highest equimolar concentration) to the Sulf-2 as a control. The reaction was carried out in a 50 μl volume, and aliquots were collected at 0, 0.5, 1, 2, and 4 h. The substrate and product(s) were quantified by ion exchange HPLC with UV detection. All assays were performed in triplicate. Half-maximal inhibitory concentration (IC50) of LG3BP on Sulf-2 activity was measured by performing the assay with a fixed amount of Sulf-2 (250 ng/ml), and serial dilution of LG3BP (0.16–20 μg/ml), using a 2h incubation time. The enzymatic reaction kinetics was measured using a fixed amount of Sulf-2, three different concentrations of LG3BP (1.875, 5, and 9.375 μg/ml), and serial dilution of the substrate (3.4–218 μM).

### Spheroid Assay

The spheroid assays using SCC35 and HNCAF61137 cells were performed as described (27). Briefly, the spheroids were formed by co-culturing the cells for 1 day in ultra-low attachment 96-well round bottom plates and were embedded in 50 μl of growth factor reduced Matrigel. Then 100 μl medium with/without LG3BP (100 ng/well or 1 μg/well; six replicates per concentration) was gently added on top of the Matrigel in each well. Spheroids were cultured for 5 days and imaged on day 1 and day 5 using Olympus IX71 inverted microscope. Digital images were analyzed for spheroid area, inverse circularity, protrusion, and endpoint measurements using INSIDIA 2.0 software (30, 31). It comprises spheroid segmentation and analysis of binarized and grayscale images. Binarized image parameters include morphological features shape extraction of spheroid boundary and quantification of maxima on the boundary profile. The data is used to calculate the number of protrusions as well as the quantification of endpoints on skeletonized spheroids. Inverse circularity is a shape parameter of the spheroid, where circularity ranges from a value of 0 to one based on the absolute circle taken as one, and with increased distortion, the number decreases. GraphPad Prism Software (v 10.1.1) was used to perform statistical analysis and data visualization. Mean and SD were calculated, and two-way ANOVA with Tukey was performed to determine statistical significance between data groups.

## Results

### Identification of Sulf-2 Interacting Proteins

HNSCC cell lines SCC35 and CAL33 (human squamous cell carcinoma cell lines derived from hypopharynx and oral cavity tumors, respectively) conditioned media was used as the starting material for immuno-affinity purification (IP) of the Sulf-2 protein in complex with its interacting partners (Supplemental Fig. S3). Sulf-2 specific monoclonal antibodies (32) bound to magnetic beads were used to pulldown the target protein and the co-purified proteins were identified by LC-MS/MS analysis. The identified proteins in these samples including proteins detected in parallel negative control are listed in Supplemental Table S1. We excluded the proteins of bovine origin and those from the contaminant database; and the results from SCC35, CAL33, and negative controls were analyzed (Supplemental Table S2). Gene ontology cellular component enrichment analysis (using string-db.org) showed that approximately 60% of the proteins in anti-Sulf-2 pulldown samples (Supplemental Table S2*E*) have extracellular region assignment (GO:0005615, FDR 5.30e-14). As expected, the Sulf-2 protein was only identified in specific mAb pulldowns but not in antibody isotype-negative control. Six of the proteins identified in Sulf-2 pulldown but not in negative control are components of collagen-containing extracellular matrix that are likely interacting partners. Of the candidates, Sulf-2 and LG3BP were consistently identified in all replicate experiments (Supplemental Table S1), including IP samples prepared under high stringency wash with 200 mM NaCl (results not shown). From these results, we infer that the Sulf-2 protein interacts with LG3BP *in vivo*.

### Validation of the Sulf-2 and LG3BP Interaction

**Fig. 1:**
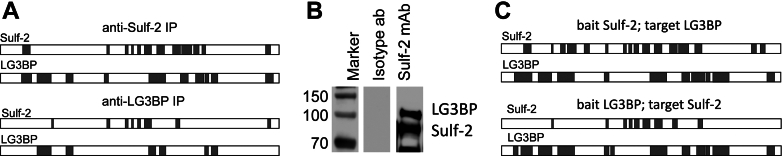
**Identification of proteins in affinity pulldown samples.***A*, LC-MS/MS analysis identified Sulf-2 and LG3BP proteins by multiple peptide matches (shaded region) in IP samples from HEK293T culture media using anti-Sulf-2 or LG3BP mAbs as indicated. *B*, Western blot analysis of the IP samples prepared from HEK293T culture using anti Sulf-2 mAb, and isotype antibody as negative control. The blot was probed with anti-Sulf-2 and LG3BP mAbs. *C*, Sulf-2 and LG3BP were identified by LC-MS/MS (matched peptide indicated by shaded region) in reciprocal co-IP samples using purified recombinant proteins, the bait, and target as noted.

We generated a recombinant Sulf-2 (rSulf-2) protein in HEK293T cells using lentiviral transduction and performed affinity purification of the Sulf-2 protein from the serum-free media of the transduced cell line using Sulf-2 mAb. Reciprocally, LG3BP protein pulldown from this sample was performed using anti-LG3BP monoclonal antibody. MS analysis identified Sulf-2 and LG3BP proteins in both pulldowns (Fig. 1*A* and Supplemental Table S3, *A* and *C*). We also verified the presence of these two proteins in the pulldown sample, and not in negative control samples, by Western blot analysis (Fig. 1*B*). Furthermore, the pulldown and MS analysis of Sulf-2 and LG3BP protein with their respective mAbs was performed in parallel from the conditioned media of stably transfected rSulf-2 HEK293F cell line (see Supplemental Table S3, *B* and *D* for list of identified proteins). In Supplemental Table S4, we have listed the extracellular proteins pulled down from specific media with the target antibodies. In aggregate 18 proteins were found to be common to the anti-Sulf2 and anti-LG3BP pulldowns (Supplemental Table S4*E*), seven of which are components of collagen-containing extracellular matrix. We compared the protein candidates identified from HNSCC pulldown to those from recombinant HEK media (Supplemental Table S5). Sulf-2 and LG3BP were consistently identified in pulldowns from all the cell types which further confirms their direct interaction. It is likely that some of the other candidates, especially components of the collagen-containing extracellular matrix are part of the complex but some may be non-specific binders. Zinc-alpha-2-glycoprotein (AZGP1) is a likely interactor as it is identified in both datasets.

In addition to the abovementioned analyses, we verified the direct interaction of Sulf-2 and LG3BP *in vitro* by reciprocal co-immunoprecipitation (co-IP) experiments using purified recombinant proteins (rSulf-2, rLG3BP). The rSulf-2 protein bound to the beads (bait) was incubated with rLG3BP (target) and vice-versa in parallel (see Experimental Procedures). Following extensive washes, Sulf-2 and LG3BP were both identified by MS analysis in both co-IP experiments but not in negative controls (Fig. 1*C*). These results unequivocally confirm the binding of Sulf-2 and LG3BP.

### Effect of Chondroitin Sulfate on Sulf-2 Interaction with LG3BP

**Fig. 2:**
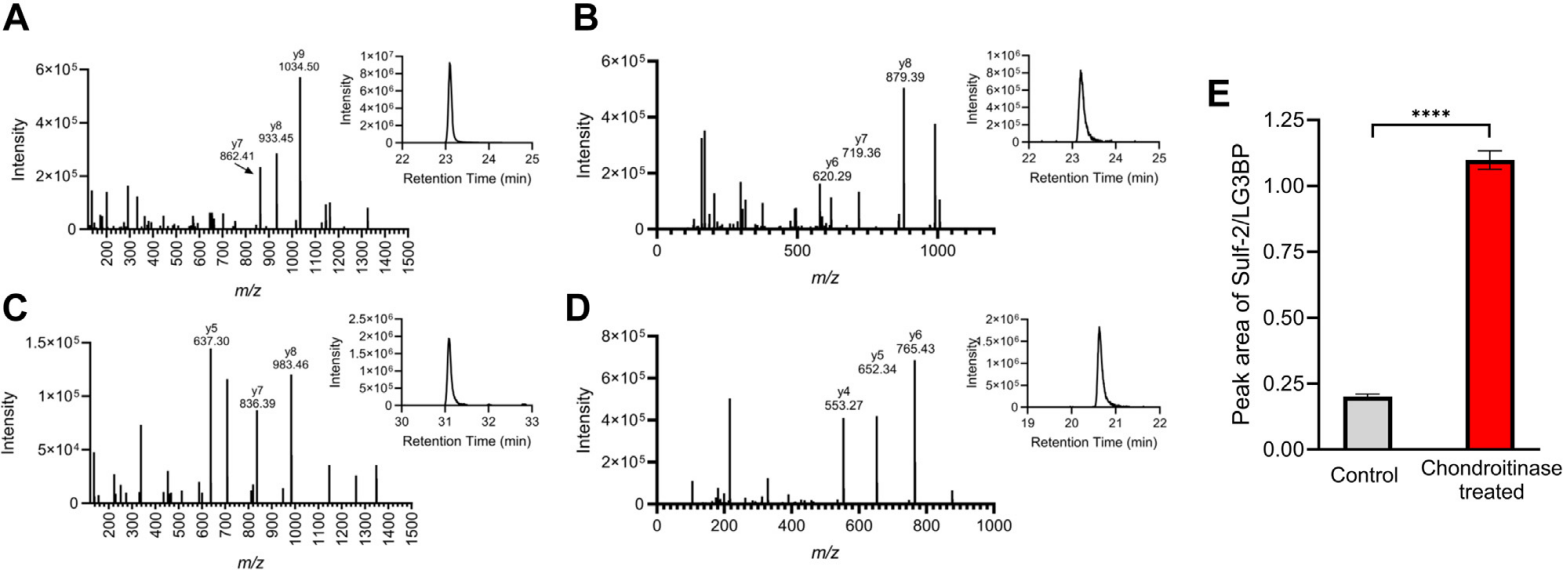
**LC-MS/MS PRM analysis of Sulf-2 and LG3BP in LG3BP mAb pulldown of chondroitinase treated and control samples.** Product ion spectra and extracted peaks using sum of indicated product ions of the following peptides: Sulf-2 target peptides (*A*) AEYQTAcEQLGQK (*z* +2, *m/z* 763.3512), (*B*) WQCVEDATGK (*m/z* 597.2664), and LG3BP peptides (*C*) YSSDYFQAPSDYR (*m/z* 799.8415, *z* 2), (*D*) SQLVYQSR (490.7616). *E*, graphical representation of the average ratio of peak areas of Sulf-2/LG3BP, showing statistically significant relative increase of Sulf-2 in the chondroitinase treated pulldown sample.

We interrogated if the chondroitin sulfate (CS) covalently attached to Sulf-2 at S583 influences its interaction with LG3BP. To assess this, we first treated the rSulf-2 media with chodroitinase-ABC, followed by affinity pulldown using anti-LG3BP mAb. A control sample was prepared in parallel under identical conditions except for the chodroitinase addition. Target LG3BP and interacting Sulf-2 proteins were identified in pulldown samples (Supplemental Table S6), and we observed that Sulf-2 had relatively higher peptide coverage in the chondroitinase-treated IP sample. The relative abundance of the Sulf-2 and LG3BP proteins in IP samples was determined by targeted LC-MS/MS-PRM of a Sulf-2, and LG3BP tryptic peptides as detailed in methods. Comparison of the peak areas of Sulf-2 normalized to LG3BP showed a significant increase in Sulf-2 binding to LG3BP upon the CS-side chain removal (Fig. 2). The results suggest that the CS side-chain influences the interaction, either directly or by modifying the Sulf-2 structure.

**Fig. 3:**
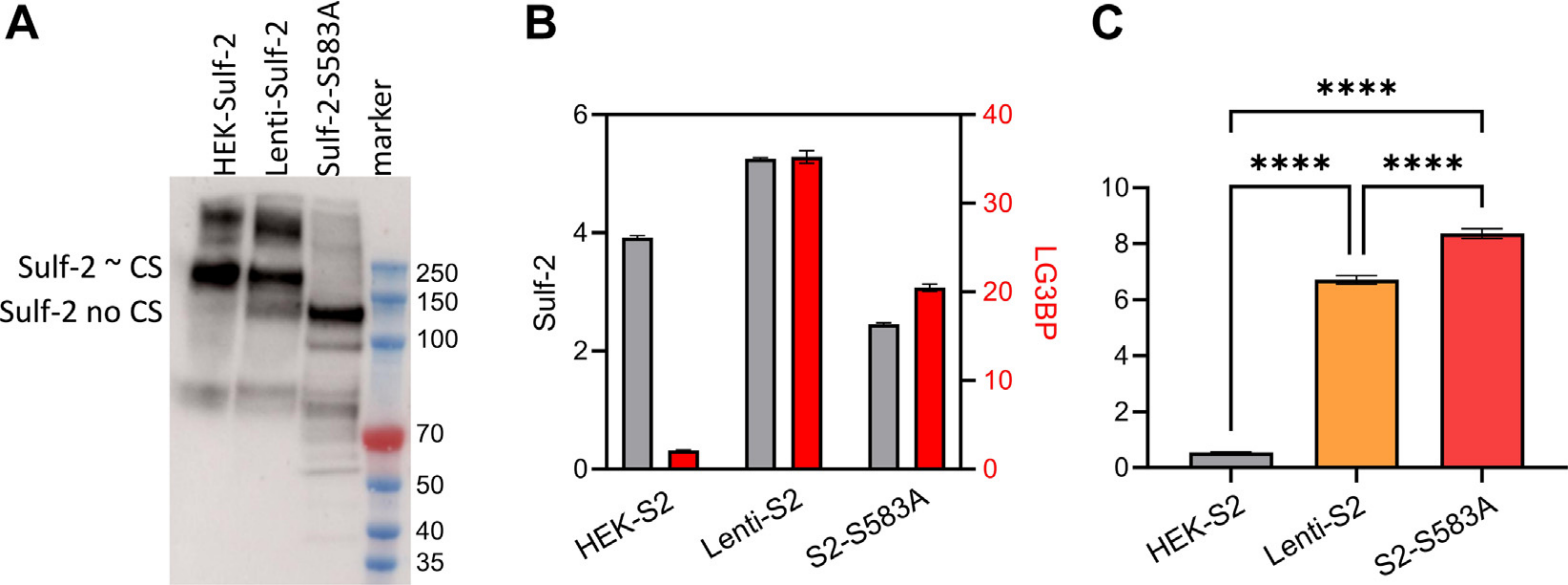
**Effect of the Sulf-2 CS chain on LG3BP interaction.***A*, Western blot analysis (non-reduced sample) showing Sulf-2 mostly chondroitinated in HEK, non-chondrotinated in S583A mutant and mixture of chondroitinated and non-chondroitinated in lenti-Sulf-2 media. *B*, LC-MS/MS-PRM quantification of Sulf-2 (*grey bars*) and LG3BP (*red bars*) in Sulf-2 mAb pulldown samples. *C*, LG3BP normalized to Sulf-2 shows a statistically significant relative increase of LG3BP binding in samples containing more non-chondroitinated Sulf-2.

To confirm the impact of the CS on binding, we prepared a S583A mutant of rSulf-2 in a HEK293F expression system as described previously (21). The lack of CS on the S583A mutant rSulf-2 was verified by native gel electrophoresis followed by Western blot analysis (Fig. 3*A*); this is in contrast to the wild-type protein which is almost completely decorated with the CS side-chain. However, a significant proportion of lentiviral expressed rSulf-2 (HEK293T cells) lacks the CS side chain. Subsequently, we determined the relative abundance of LG3BP in the complex with the S583A Sulf-2 by the LC-MS/MS-PRM assay in pulldown samples prepared with anti Sulf-2 mAb. The relative abundance of LG3BP was highest in the Sulf-2 CS mutant, followed by wild-type Sulf-2 expressed in the HEK293T lentiviral sample (which has a substantial proportion of Sulf-2 lacking the CS), and lowest in wild-type Sulf-2 expressed in the HEK293Fcell (nearly completely CS-modified) (Fig. 3*B*). The results show a statistically significant inverse correlation of the binding to the CS content of the Sulf-2 preps. The results together suggest CS chain impacts the binding of LG3BP to the Sulf-2 protein and may impact their interaction *in vivo*.

### Predicted 3D Model of Sulf2 and Sulf-2/LG3BP Complex

**Fig. 4:**
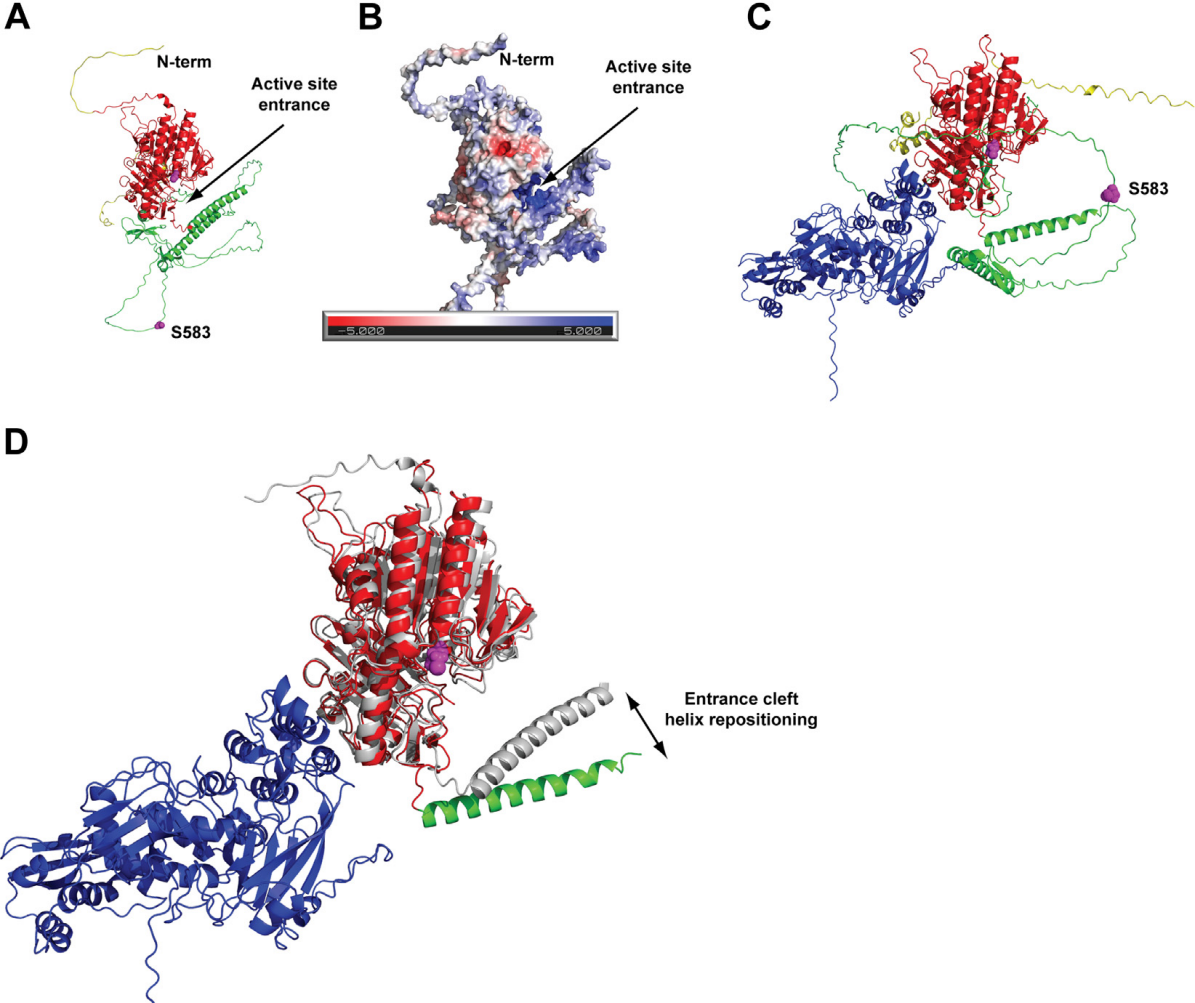
**AlphaFold model of the Sulf-2 and Sulf-2/LG3BP complex.***A*, cartoon representation of the Sulf-2 structure; the catalytic core, the hydrophilic domain, and N/C-termini are colored *red*, *green*, and *yellow* respectively. The active site Cys88 and chondroitin sulfate attachment site Ser583 are shown as magenta spheres. *B*, the electrostatic surface of Sulf-2 with the positively charged (*blue*) active-site entrance is indicated by an *arrow*. *C*, the predicted Sulf-2/LG3BP complex, LG3BP is colored blue and Sulf-2 is colored as in *panel A*. *D*, overlay of the Sulf-2/LG3BP complex (*colored*), and free Sulf-2 (*grey*). Note the repositioning of the entrance cleft helix (amino acids 684–716) upon LG3BP binding that leads to remodeling of the architecture of the active-site entrance. Disordered Sulf-2 segments were omitted for clarity.

No experimental structures are available for human Sulf-2 protein, so we performed *in silico* prediction of the 3D structure. The model can be divided into a predominantly structured catalytic core comprising amino acids 35 to 420 and 720 to 830 (Fig. 4*A*; red), and a mostly unstructured hydrophilic domain comprising amino acids 421 to 719 (green). Additionally, both N- (amino acids 1–34) and C-termini (amino acids 831–870; yellow) of the protein are predicted to be intrinsically disordered. The catalytic domain harbors Cys88 which needs to be converted to formylglycine for Sulf-2 catalytic activity. The active site is situated at the bottom of a positively charged pocket (Fig. 4*B*). It appears that the positively charged C-terminal segment (amino acids 702–713) of the hydrophilic domain is structurally adjacent to the active site pocket and provides an additional positively charged surface which could be essential for the Sulf-2 substrate binding cavity (Fig. 4*B*).

In the predicted Sulf-2/LG3BP complex, the major interaction interface comprises two helices of the LG3BP (amino acids 353–396) interacting with loops amino acids 242 to 256 and 783 to 793 within the structured catalytic core of Sulf-2 (Fig. 4*C*). Such interaction is predicted to have minimal effects on the 3D fold of the catalytic core and does not sterically block the entrance into the Sulf-2 active site. This is consistent with the observed non-competitive inhibition of the Sulf-2 activity by the LG3BP (see below). At the same time, the LG3BP binding to Sulf-2 is predicted to result in the repositioning of the C-terminal positively charged segment (amino acids 702–713) that could lead to the disruption of the overall architecture of the substrate binding cavity (Fig. 4*D*), and consequently negatively impact the Sulf-2 catalytic activity.

### Effect of LG3BP on Sulf-2 enzymatic Activity

**Fig. 5:**
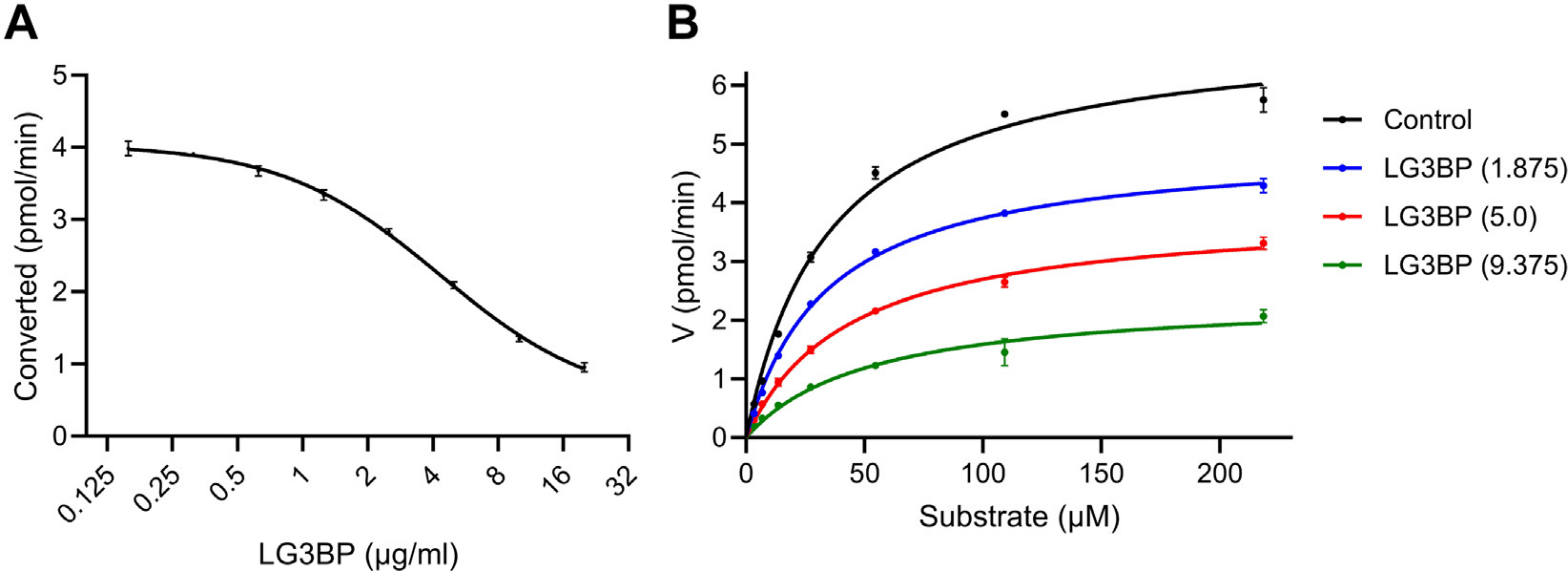
**Measurement of the Sulf-2 enzymatic kinetics with added LG3BP.***A*, IC50 measurement showing converted substrate (y-axis pmol/min) with 2-fold increasing concentration of LG3BP (x-axis) at fixed concentrations of Sulf-2 (250 ng/ml), and substrate (55 μM).*B*,Michaelis-Menten curve showing velocity of substrate conversion (y-axis), with2-fold increasing substrate concentration (x-axis), with control and added LG3BP at three different concentrations (μg/ml) as indicated in parenthesis, at a fixed concentration of Sulf-2 (250 ng/ml).

Sulf-2 enzymatic activity was measured in the presence and absence of LG3BP using a 2S2-6S4 synthetic oligosaccharide substrate as described recently (21). Purified rSulf-2 was incubated with varying concentrations of purified rLG3BP followed by the addition of a fixed concentration of the 2S2-6S4 substrate. The desulfation of the substrate by Sulf-2 was inhibited by LG3BP in a dose-dependent manner (Supplemental Fig. S4). Serial dilution of LG3BP with constant Sulf-2 and substrate showed IC50 of 4.3 μg/ml *in vitro* (Fig. 5*A*). Then the reaction was performed at three different concentrations of LG3BP (1.875, 5.0, 9.375 μg/ml) with increasing substrate concentration to measure the enzyme kinetics (Fig. 5*B*). We observed the Vmax of control, added LG3BP 1.875, 5.0, and 9.375 μg/ml, respectively, 7.1 ± 0.2, 5.7 ± 0.1, 3.6 ± 0.2, and 1.8 ± 0.2; and Km, respectively, of 39.9 ± 1.1, 43.5 ± 0.7, 37.66 ± 2.3, and 27.9 ± 5.3. The slope, respectively, was 5.6, 7.7, 10.4, and 15.6; with Y-intercept at 0.14, 0.18. 0.28, and 0.57, and X-intercept at −0.03, −0.02, −0.03, −0.04. This showed that LG3BP non-competitively inhibits Sulf-2 enzymatic activity. The result confirms a direct interaction of the Sulf-2 and LG3BP proteins and shows that the interaction has functional consequences *in vitro*, which could impact the Sulf-2 activity in model systems and *in vivo*.

### Inhibition of Cancer Cell Invasion by LG3BP in a Spheroid Model

**Fig. 6:**
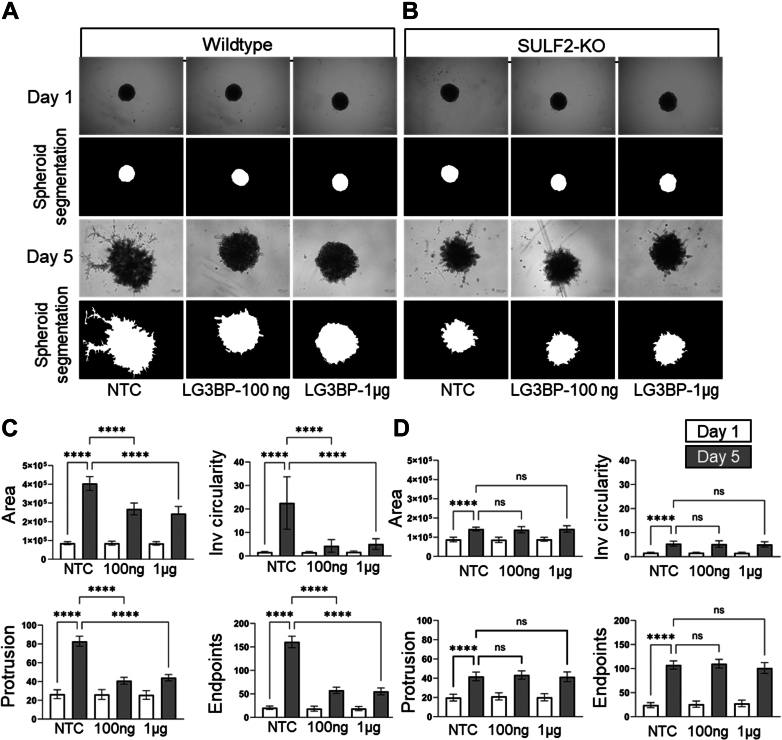
**Effect of LG3BP addition on the invasion of SCC35 spheroids in Matrigel.***Upper left panel* (*A*) shows assays using WT SCC35 cells co-cultured with HNCAF61137 cells, *right panel* (*B*) shows SCC35 SULF2-KO (knock out) cells co-cultured with HNCAF61137 cells. Representative bright field images with phase contrast (*top row*) and masked grayscale images generated with INSIDIA (*bottom row*) of the co-culture spheroids grown in Matrigel and imaged on day 1 and day 5. Scale bar 200 μm. *Lower panel* graphical representation of spheroid invasion (day 1 open box, day 5 *shaded box*) measured without LG3BP (NTC) and with two concentrations of LG3BP (100 ng and 1 μg) as indicated in the following co-cultures: (*C*) SCC35 wild-type spheroids; (*D*) SCC3 SULF2-KO spheroids. Results are reported as total area, inverse circularity, protrusions, and endpoints. Bars indicate mean ± SD with statistical significance evaluated using two-way ANOVA with post hoc Tukey’s test. *p*-values < 0.05 are considered significant, *p*-values <0.0001 are represented by ∗∗∗∗.

We recently reported a Sulf-2-dependent spheroid invasion model consisting of a co-culture of SCC35 cancer cells with a primary cancer-associated fibroblast (HNCAF37) in Matrigel (27). We have shown that Sulf-2 KO SCC35 cells invade Matrigel in this system significantly less than the wild-type SCC35 cells. In this study, we used the same model to assess how an exogenous addition of LG3BP to the spheroid model affects cancer cell invasion (Fig. 6). The control spheroids significantly invaded Matrigel by day 5, as expected. We observed a significant reduction in the invasion phenotypes on day 5 in the LG3BP-treated spheroids. Compared to the no treatment control (NTC), the area of the spheroids was reduced to 66%, inverse circularity to 19%, protrusion to 49%, and endpoints to 36% with the addition of 100 ng LG3BP. No significant differences were observed between day 5 spheroids treated with 100 ng or 1 μg of LG3BP, indicating saturation of the binding already at 100 ng. Subsequently, we used SCC35 Sulf-2 KO cell in the spheroid assay and we observed a reduced influence of the LG3BP. The NTC Sulf-2 KO spheroids invaded Matrigel less efficiently than the NTC Sulf-2 wild-type spheroids (reduction of area to 35%, inverse circularity to 24%, protrusion to 51%, and endpoints to 67%), as expected, but the addition of LG3BP did not significantly alter the outcome in the Sulf-2 KO spheroids. Thus, the addition of LG3BP affected the invasion of the wild-type SCC35 cells into Matrigel in a Sulf-2-dependent manner. This confirms that the inhibition of Sulf-2 enzymatic activity could have functional consequences *in vivo*.

## Discussion

Heparan-6-*O*-endosulfatases Sulf-1 and Sulf-2 are secreted extracellular enzymes overexpressed in many types of cancer (12, 13, 14, 16, 17, 18, 33). The Sulf-1 protein is upregulated in the tumor tissues, but in general, is not detectable in cancer cell lines. Sulf-2 is secreted in the tumor tissues primarily by cancer cells while Sulf-1 is supplied to the tumor microenvironment by CAF cells. Sulf-2 is considered oncogenic (19) and we have shown that Sulf-2 is associated with poor survival outcomes in HNSCC (16, 17). Although Sulf-1 and Sulf-2 share many common features and *in vitro* enzymatic activity, only Sulf-2 is a proteoglycan modified by a CS chain reported to affect its enzymatic activity (20, 26). However, it remains unknown what other factors regulate the activity of the Sulf enzymes *in vivo*. We suspected that the activity of Sulf-2 depends on interacting regulatory proteins, but virtually nothing is known about its activity-modifying interactions. In this study, we therefore searched for Sulf-2 interacting proteins that modify its enzymatic activity.

We identified LG3BP in Sulf-2 pulldown from the conditioned media of two different HNSCC cell lines under high stringency washes. The association of the two proteins was further verified by complementary pull down of Sulf-2 and LG3BP with their respective mAbs from the conditioned media of cell lines overexpressing a recombinant Sulf-2 protein (Fig. 1). It is possible that the LG3BP associates with Sulf-2 in a complex with other proteins because we identified other candidates collagen-extracellular matrix component proteins (LG3BP is a component of this ECM) in the pulldown samples after subtraction of a negative pull-down control and common contaminants. However, LG3BP interacts with Sulf-2 directly, independent of the other proteins (see below). We therefore focused on characterizing the Sulf-2 interaction with LG3BP and evaluated its impact on Sulf-2 enzymatic activity.

To confirm that the Sulf-2 and LG3BP bind directly, we performed reciprocal co-IP experiments using purified recombinant proteins (Fig. 1*C*). Our results show clearly a direct interaction of the two proteins and we infer that they likely directly interact *in vivo*. However, it is interesting to note that the interaction depends on the CS modification of Sulf-2. Treatment of Sulf-2 with chondroitinase ABC increases relative amount of Sulf-2 in LG3BP pulldown (Fig. 2). In addition, we observed in Sulf-2 pulldown experiments from our recombinant cell cultures that LG3BP binds weaker to the chondroitinated Sulf-2 (Fig. 3). We showed by targeted MS assays that the relative abundance of LG3BP in the pulldown samples is inversely related to the chondroitination of Sulf-2. To validate this observation, we generated a mutant Sulf-2 that lacks the CS-chain and we found that it pulls down LG3BP more efficiently (Fig. 3). Thus, the CS-side chain of Sulf-2 may be an important modifier of this interaction, and the degree of chondroitination of the Sulf-2 enzyme in different cells and tissues needs to be further examined together with the structure of the CS chain.

LG3BP is a relatively abundant secreted protein ubiquitously present in human tissues and detectable in serum. It is a multi-function glycosylated protein, an abundant component of cancer-derived EVs, and a key regulator of cancer-stroma interactions (34). An association of high LG3BP in tumors with poor survival has been reported. It has been described as a biomarker and as a therapeutic target (34, 35). Overall, it is a substantially more abundant protein than the Sulf-2 enzyme. It is also nearly 100 times more abundant in serum than galectin 3, its well-recognized binding partner (36). LG3BP is detected in human serum at concentrations of approximately 3 μg/ml, is further elevated in the sera of cancer patients, and could have even higher titers in the tumor microenvironment (36, 37). It is also an abundant protein in the secretomes of all our HNSCC cancer and CAF cell lines which further supports our assumption that it interacts with Sulf-2 *in vivo*.

We assessed the functional significance of the Sulf-2 interaction with LG3BP *in vitro* by an enzymatic assay (Fig. 5). We observed that LG3BP at 4.3 μg/ml inhibited Sulf-2 activity by 50% *in vitro*. Our results show that LG3BP non-competitively inhibited Sulf-2 activity in a concentration-dependent manner at concentrations and inhibitor/enzyme ratio’s plausible *in vivo*. This observation is consistent with our structural model (Fig. 4) predicting that LG3BP does not directly inhibit substrate binding and minimally influence the 3-D fold of the Sulf-2 catalytic core but could disrupt the architecture of the substrate binding cavity. The formation of Sulf-2/LG3BP complex in biological samples might be affected by additional factors not observed when we compare the interaction of two purified recombinant proteins; we have already shown the influence of the CS chain which was not yet evaluated sufficiently *in vivo*. However, it is very plausible these two proteins have direct interaction *in vivo*. Similarly, the impact of LG3BP on Sulf-2 activity *in vivo* will need further evaluation even though our results show that the interaction is a plausible regulator of the Sulf-2 enzymatic activity. This is further supported by our spheroid co-culture models. We have shown that the addition of purified recombinant LG3BP to the spheroid culture media inhibited invasion of the SCC35 cells into Matrigel (Fig. 6). Invasion of the Sulf-2 KO SCC35 cells is significantly reduced compared to wild-type cells, and the addition of LG3BP had no additional effect, which is similar to a previously observed effect of a Sulf-2 inhibitor in this model system (27). Thus, we infer that LG3BP directly interacts with Sulf-2 and inhibits the Sulf-2-dependent cancer cell invasion.

In summary, we have shown that the Sulf-2 enzyme interacts directly with LG3BP. This interaction inhibits the Sulf-2 enzymatic activity and is modified by the CS modification of Sulf-2. We propose that this interaction represents a plausible *in vivo* regulatory mechanism of the Sulf-2 editing of heparan 6-*O*-sulfation. The results strongly support the need for further evaluation of this regulatory interaction in relevant physiological and pathologic conditions.

## Data Availability

The mass spectrometry data have been deposited to the jPOST repository. The accession numbers are JPST003049 for jPOST and PXD051711 for ProteomeXchange.

## Supplemental data

This article contains supplemental data.

## Conflict of interest

The authors declare that they have no known competing financial interests or personal relationships that could have appeared to influence the work reported in this paper.
