# Supplementary material for: Galectin-3-Binding Protein Inhibits Extracellular Heparan 6-O-Endosulfatase Sulf-2
Source: Mol Cell Proteomics. 2024 Jun 1;23(7):100793. doi: 10.1016/j.mcpro.2024.100793 (PMC11259796; doi:10.1016/j.mcpro.2024.100793)
Supplement: Supplemental Figures [file mmc1.pdf]

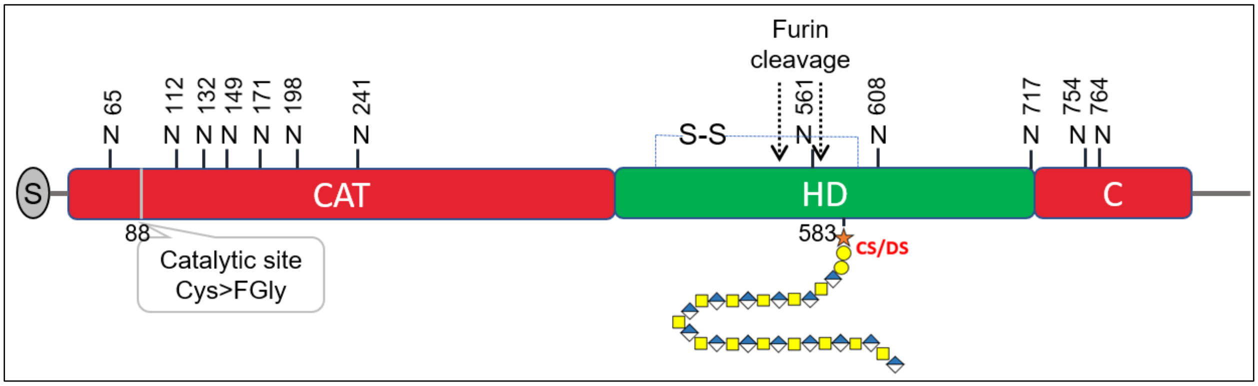

Supplementary Figure 1. Schematic representation of Sulf-2 protein domains and PTMs. Signal peptide (S), catalytic domain (CAT), hydrophilic domain (HD), and C-terminal domain (C); and enzymatic active site (FGly), N-glycosylation sites (N), chondroitin sulfate binding site, furin cleavage sites, and di-sulfide bridge are indicated. In 3D fold, the structured catalytic core is formed by CAT and C together.

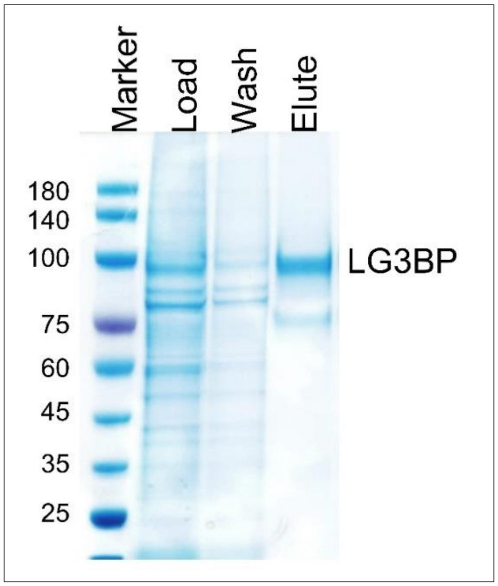

Supplementary Figure 2. Coomassie stained SDS-PAGE of Ni^{2+} affinity purified LG3BP (lane-Elute). Protein profile of starting conditioned media (Load) and from washing step (Wash) is shown.

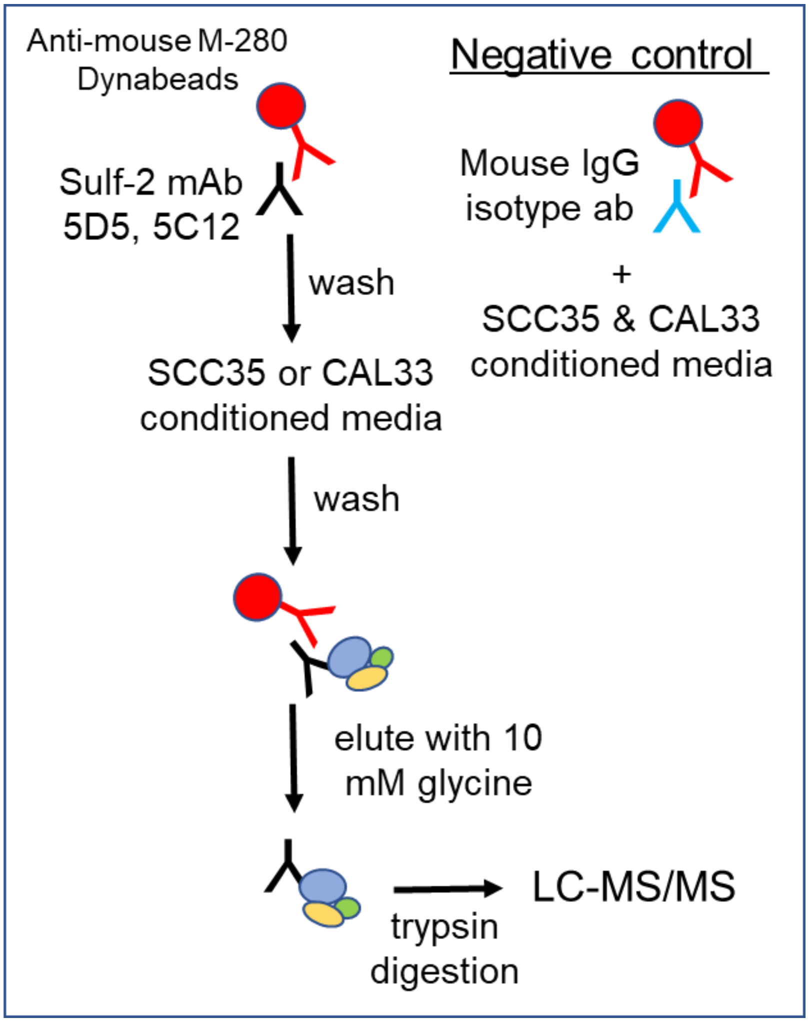

Supplementary Figure 3. Schematics of the affinity pull-down method of Sulf-2 and associated proteins using monoclonal antibodies.

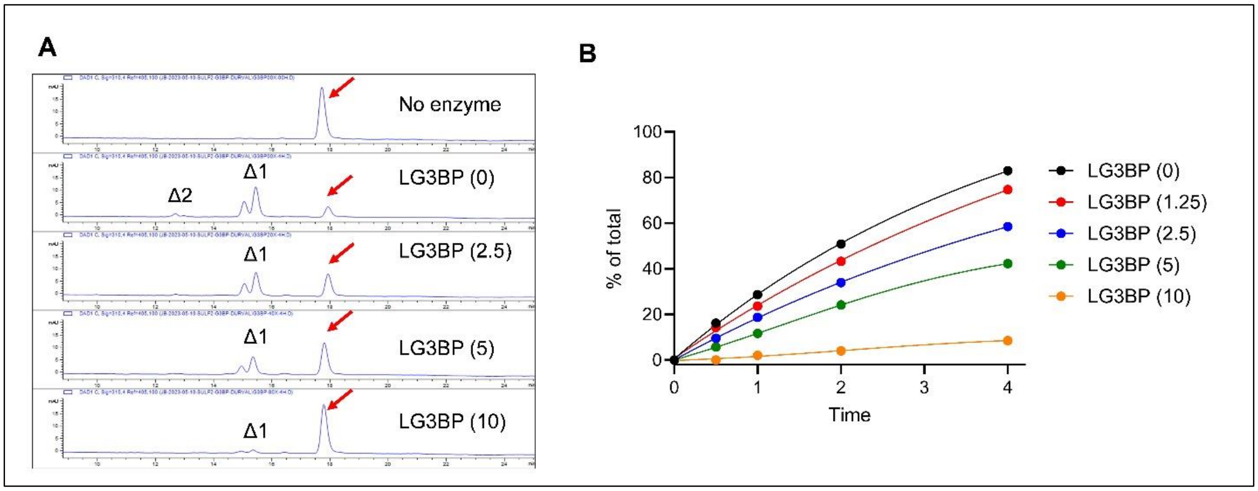

Supplementary Figure 4. Measurement of the Sulf-2 enzymatic activity with LG3BP added at increasing concentrations. The concentration of LG3BP µg/ml is indicated in parentheses. (A) Representative chromatograms showing the substrate 2S2-6S4 (indicated by red arrow) and product (Δ1, Δ2) peaks where the products with 1 or 2 sulfates removed decrease with increasing LG3BP additions. (B) Percent of converted substrate over a 4h time course with fixed concentration of Sulf-2 (250 ng/ml).
